# *Arabidopsis* bZIP68 Modulates Flowering Time Potentially with Chromatin and Stress-Responsive Regulators

**DOI:** 10.3390/biology15171525

**Published:** 2026-09-03

**Authors:** Chaelin Park, Kyoung Rok Geem, Bae Young Choi, Jaewook Kim

**Affiliations:** 1 Department of Biology Education, Korea National University of Education, Cheongju 28173, Republic of Korea; chaelin6023@knue.ac.kr; 2 Department of Biological Sciences, Gyeongkuk National University, Andong 36729, Republic of Korea; krgeem@gknu.ac.kr; 3 School of Liberal Arts and Sciences, Korea National University of Transportation, Chungju 27469, Republic of Korea

**Keywords:** bZIP68, bioinformatic analysis, flowering time, abiotic stress

## Abstract

In *Arabidopsis thaliana*, the physiological functions and regulatory network of the transcription factor bZIP68 remain poorly understood. To investigate its potential functions and regulome, we integrated multiple publicly available omics datasets, including protein–protein interaction and co-expression networks, transcriptomic data from *bzip68* mutants, bZIP68 ChIP-seq data, and ChIP-seq/DAP-seq and transcriptomic datasets for candidate regulatory factors. These integrated analyses suggested that bZIP68 is involved in floral morphology, flowering regulation, and responses to abiotic stress. Notably, SWI3D, MINU2, and CAMTA1 were identified as highly probable components of the bZIP68 core regulome. Subsequent transcriptomic network analysis revealed their putative signaling cascades. To validate the predicted role of bZIP68 in flowering regulation, we analyzed five independent homozygous EMS mutant (HEM) lines. All five mutant lines exhibited altered flowering phenotypes compared to the wild type. In conclusion, this study establishes an integrative framework for identifying the bZIP68 regulatory network and uncovers its novel physiological role in modulating flowering.

## Simple Summary

The physiological function of the transcription factor bZIP68 is relatively unknown, although it has been implicated in redox-related processes and photomorphogenic regulation. Through comprehensive bioinformatic analyses, we identified a potential association between bZIP68 and the regulation of flowering and responses to abiotic stress. Furthermore, we revealed several candidate factors that may function together with bZIP68. Phenotypic evaluation of five potential independent mutant lines revealed altered flowering phenotypes compared to wild-type plants. Overall, our findings elucidate a novel physiological function of bZIP68 in flowering regulation and its potential binding partners that may contribute to its function.

## 1. Introduction

As sessile organisms, plants, including *Arabidopsis thaliana*, rely on numerous transcription factors for their physiological responses or phase transitions [1,2,3]. *Arabidopsis thaliana* is one of the most extensively studied model plants, with a high-quality reference genome, comprehensive gene model annotation, and extensively curated resources linking genes to molecular functions, biological processes, expression patterns, and mutant phenotypes [4,5,6,7]. Yet, many transcription factors remain less studied, and concise physiological functions have not been annotated. The absence of obvious mutant phenotypes may result from complex genetic interactions and functional redundancy among transcription factors, particularly among closely related members of the same family or subfamily [8,9,10].

bZIP68 was first reported to be a basic region leucine zipper transcription factor whose activity is regulated in a redox-dependent manner [11]. Later, bZIP68 was shown to balance stress tolerance with growth [12]. In detail, *bzip68* mutation leads to retarded root growth under low-light or low-temperature conditions in a 3% sucrose medium [12]. Another line of evidence for the physiological contribution of bZIP68 suggested its involvement in photomorphogenesis [13]. This was confirmed by its redundant function with GBF1 (G-box-binding factor 1) and bZIP16 [14]. In detail, bZIP68 redundantly regulated cotyledon opening [14]. Also, *bzip68-1* was shown to have a delayed flowering phenotype, while *bzip68-2* did not [12]. bZIP68 was shown to be transported into the nucleus more extensively in H_2_O_2_-treated conditions [12]. In plants, H_2_O_2_ is a pleiotropic player in various stress conditions and developmental processes. In drought stress conditions, abscisic acid (ABA) induces H_2_O_2_, which activates the calcium channel and induces stomatal closure [15]. Recently, H_2_O_2_ has been considered a signaling molecule, since HPCA1 (H_2_O_2_-Induced Ca^2+^ Increases 1) was discovered to be a receptor associated with stomatal closure [16]. Stomatal developmental processes rely on H_2_O_2_ molecules as well, not only under stress conditions [17]. In the light-induced stomatal opening process, H_2_O_2_ was observed to accumulate and be associated with other processes [18]. In addition to the stomata, root growth and cell division are deeply associated with H_2_O_2_, including ROS species [19,20]. These observations suggest that bZIP68 might exert broader physiological influence than currently established, underscoring the need for a comprehensive functional characterization.

Bioinformatic analysis with an enormous database can reveal the unseen regulome and physiological functions for any genes of interest. AraNet is a gene-function association network based on co-expression, protein–protein interaction (PPI), and comparative genomic data [21]. Using this analysis, *AT1G80710* (*DROUGHT SENSITIVE 1*) was revealed to be associated with drought tolerance, and *AT3G05090* (*LATERAL ROOT STIMULATOR 1*) was associated with secondary root development [21]. Comprehensive co-expression network analysis with seed-to-seedling phase transition transcriptome revealed AtHB13 as a potent regulator, and its mutant was found to have elongated root length [22]. A similar approach was adapted with Heuristic Cluster Chiseling Algorithm (HCCA) and revealed six genes associated with early seedling development [23]. A combination of gene expression ranking and co-expression network analysis revealed a large-scale identification of heat-stress-associated genes, which were subsequently validated through mutant analysis [24]. Consequently, most of these approaches have focused on discovering novel genes responsible for targeted physiological responses. Although multi-omics network-based functional annotation has been attempted, these predictions have rarely been integrated with experimental validation using physiological characterization of mutant or transgenic lines [25].

Although a few physiological roles of bZIP68 have been studied, its contributions to complex processes such as flowering, as well as other potential functions, remain poorly characterized [12]. Here, we adapted a vast multi-omics approach to reveal the functional hints and regulome of bZIP68, following an approach similar to a previous study [25]. We performed PPI network analysis and co-expression analysis to reveal bZIP68 directly associated genes. These genes were associated with the flowering process, abiotic stress, and other hormone signaling pathways. Re-analysis of *bzip68* mutant transcriptome and Chromatin immunoprecipitation-sequencing (ChIP-seq) of bZIP68 confirmed the hint revealed by directly associated genes. Our integrative analysis of publicly available *A. thaliana* RNA-seq, ChIP-seq, and DNA affinity precipitation sequencing (DAP-seq) datasets revealed more than 60 factors indirectly associated with bZIP68. Of those, three factors (SWI3D, MINU2, and CAMTA1) were shown to be highly associated with bZIP68 through comparative analysis using publicly available RNA-seq data derived from the respective mutants of these factors. Consequently, these analyses indicated that bZIP68 would be associated with abiotic stress, flower development, and defense responses. To confirm the physiological function of bZIP68, we analyzed five homozygous EMS mutagenesis (HEM) lines, all of which showed altered flowering time accompanied by abnormal flower morphology compared to the wild type. Taken together, our study provides comprehensive bioinformatic evidence elucidating the role of bZIP68, which was further validated by physiological experiments.

## 2. Materials and Methods

### 2.1. Phylogenetic Analysis of bZIP68

bZIP68 (AT1G32150, Uniprot ID: Q84LG2) was queried onto Araport11 proteome database with BLASTp 2.12.0+ to identify homologs [6,26]. In detail, percent identity and e-value were set to 90% and $1\times 10^{-10}$, respectively. Then, all the homolog sequences were aligned with MAFFT v7.490 [30 October 2021] with the default option [27]. Alignment data were trimmed with trimAl v1.5.rev0 build [27 May 2024] with automated1 option [28]. The trimmed alignment was then analyzed with IQ-TREE multicore version 2.0.7 [29]. Branch support was assessed using 1000 SH-aLRT replicates and 1000 ultrafast bootstrap replicates. The resulting tree file was visualized with FigTree v1.4.5 [30].

### 2.2. Network Analysis of bZIP68 to Reveal Direct Associations

The Uniprot ID of bZIP68 was queried in GeneMania v3.5.3 in Cytoscape v3.10.2 with two approaches as follows [31,32]: 1. Searching only physical interactions and predicted networks by retrieving the top 200 related genes across all attributes using Gene Ontology—Biological Process (GO-BP) weighting. 2. Searching only co-expression networks with the same approach as 1. Then, DAVID GO was applied for 200 genes and those with functional annotations (GO-BP) were used for the following analysis [33]. For final visualization, all the query genes were re-analyzed using GeneMania v3.5.3 in Cytoscape v3.10.2 to generate a consolidated network including physical interactions, predicted networks, and co-expression networks, without searching for additional related genes [31,32].

### 2.3. Transcriptome Analysis

The target transcriptome was selected based on the physiological background of the *bzip68* mutant transcriptome, using the following criteria: 7- to 14-day-old seedlings or aboveground tissues. All the read files were downloaded with sratoolkit version 2.11.3. The software is available online: https://github.com/ncbi/sra-tools (accessed on 15 October 2024). Reads were polished with Trimmomatic v0.39 and PRINSEQ-lite 0.20.4 [34,35]. Polished reads were mapped onto the Araport11 transcriptome with BOWTIE2, and mapped reads were counted and TMM-normalized with RSEM v1.3.1 [6,36,37]. Then, EdgeR version 3.16.5 was used to compute the negative binomial dispersion across conditions for differential gene expression analysis [38]. Differentially expressed genes (DEGs) were identified using a false discovery rate (FDR) adjusted for *p* < 0.01 [39]. Visualization of gene expression via heatmap was performed with TB-tools [40].

Association score between the public transcriptome and *bzip68* mutant transcriptome was calculated with Spearman’s ranked correlation by following the order [41]: 1. Calculate average Transcripts per million (TPM) of each sample. 2. Cut off all the genes with lower expression (less than 1 transcript). 3. Calculate log_2_[Fold Change (FC)] and normalize lower and upper limits. In detail, -infinite value was set to 0 and infinite value was set to 100,000. 4. Transfer to a rank table and calculate the absolute correlation value. Visualization of the association score was done with ggplot2 from R or Microsoft excel [42]. Based on the association score, we defined bZIP68 core regulome by association score > 0.4. By this criteria, we could identify three members and denote as bZIP68 core regulome.

Potential regulatory modules were investigated with GENIE3 v1.24.0 [43]. After identifying core regulome, we determined 323 co-DEGs which were shared with all four-transcription regulator mutant transcriptome (bZIP68, SWI3D, MINU2 and CAMTA1). Then, transcription factors were defined as a regulator and the other genes were defined as network member genes and subjected to GENIE3. GENIE3 was run using default parameters to calculate the regulatory weight matrix. Because GENIE3 importance scores provide a relative ranking of putative regulatory interactions rather than a statistical significance measure, we retained the top 1% of edges ranked by weight to focus on the strongest predicted regulatory relationships, following percentile-based filtering approaches used in previous GRN studies [44,45]. Among them, we selected the regulators with more than 7 node members. Then, the edges and nodes were exported and visualized in Cytoscape [31].

### 2.4. ChIP-Seq and DAP-Seq Analysis

The read files were downloaded using sratoolkit version 2.11.3. The software is available online: https://github.com/ncbi/sra-tools (accessed on 10 December 2024). Reads were polished with Trimmomatic v0.39. Then, cleaned reads were mapped onto the Araport11 genome sequence with BWA 0.7.17-r1188 and processed with samtools version 1.13 [46,47]. The resulting sorted bam files were processed with HOMER v5.1 with default settings and the tair10 option [48].

Peak tables were converted to BED format and standardized as 500 bp windows centered on each peak midpoint with bedtools v2.30.0 [49,50]. Pairwise peak overlap between bZIP68 and other transcription factor datasets was calculated using shared peak counts, directional overlap fractions, Jaccard similarity, overlap coefficient, and Fisher’s exact test with Benjamini–Hochberg correction [49,51]. In detail, when two peaks overlapped by more than 250 bp, they were defined as a shared peak region and selected for further analysis [52]. A minimum overlap fraction was used as an effect-size criterion, while statistical enrichment of genomic co-localization was independently assessed using Fisher’s exact test with FDR correction. To evaluate broader co-occupancy patterns, all-to-all bidirectional overlap among transcription factor peak sets was visualized as dot heatmaps [42,49,53]. For spatial profiling, bZIP68 peak midpoints were used as anchor points, and candidate transcription factor peak occupancy was calculated across fixed genomic bins surrounding the anchor [53]. bZIP68 peaks substantially overlapping candidate transcription factor peaks were extracted and annotated to nearby genes using HOMER annotatePeaks.pl [48]. Indicated factors were then further analyzed with association scores [41].

### 2.5. Functional Annotation

DAVID GO was applied for gene ontology term identification [33]. GO analysis for regulome downstream genes was performed using an Expression Analysis Systematic Explorer (EASE) score threshold of 0.5, while all remaining parameters were set to default options. Then, gene set enrichment analysis (GSEA) was applied to further indicate functional annotation [54]. Visualization of GO and GSEA results was performed using ggplot2 [42].

### 2.6. Plant Growth Conditions

*Arabidopsis thaliana* seedlings were grown on tap-water-soaked paper towels under continuous light conditions for 7 days. Then, seedlings were transferred to pre-soaked soil, and the pots were wrapped for an additional 10 days. Plants were grown under long-day conditions (16 h light and 8 h dark) at a light intensity of 17.4827 W/m^2^ using white light for the indicated period. All the *A. thaliana* seeds were in the Col-0 (wild type) or HEM (Homozygous EMS Mutant lines, ES1M5S11061, ES1M5S10226, ES1M5S11015, ES1M5S06062, and ES1M5S10019). To observe the general growth features, plants were grown on soil at 22 °C.

## 3. Results

### 3.1. Identification of bZIP68-Interactomes and Co-Expressed Gene Network

**Figure 1:**
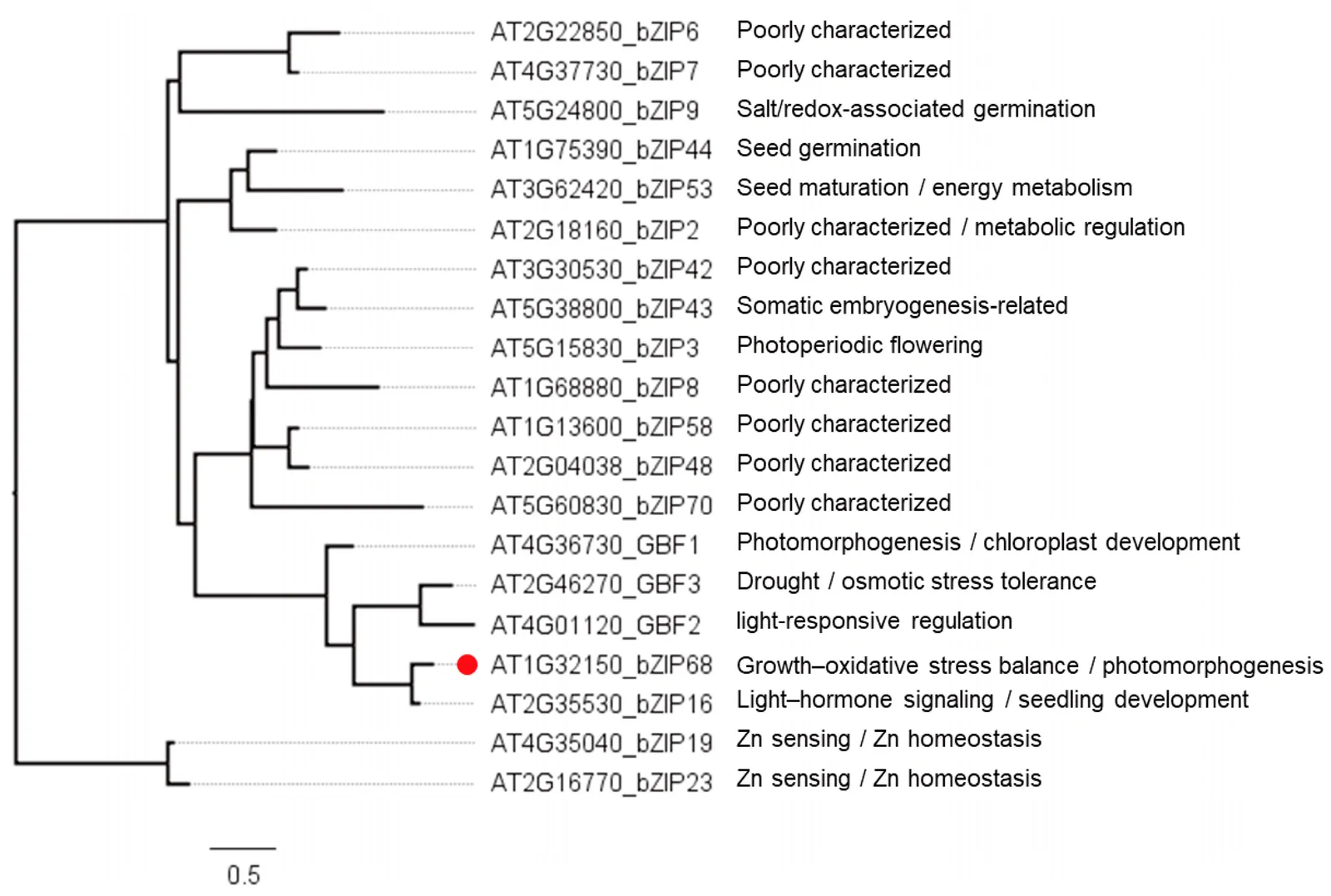
Phylogenetic tree of bZIP68 and its nineteen homologs. Red dot indicates the position of bZIP68. bZIP19 and bZIP23 was set as an outgroup and known physiological functions for each bZIP member were indicated in the right side. The scale bar indicates the number of amino acid substitutions per site.

The bZIP transcription factor family comprises numerous members in most plant genomes, many of which arose through gene duplication and may retain partially overlapping functions [8,55]. Genes occupying isolated phylogenetic positions or lacking close paralogs are more likely to perform non-redundant or lineage-restricted functions, whereas recently duplicated family members with high sequence and expression similarity tend to retain overlapping functions and compensate for one another [56,57,58,59]. Thus, we wanted to reveal the phylogenetic relationship of bZIP68 in *A. thaliana* (Figure 1). Eighteen members were identified as intraspecies homologs, and among them, bZIP16 was shown to be the closest sister member of bZIP68 (Figure 1). In addition to bZIP16, GBFs were also shown to be closely related to bZIP68. Considering the known physiological functions of these homologs, photomorphogenic function of bZIP68 was highly suggested and subsequently confirmed (Figure 1). However, closely related members tend to possess their own original functions, such as chloroplast development of GBF1 or drought/osmotic stress tolerance of GBF3 (Figure 1). This suggests that bZIP68 may not only share common functions with its sister members (photomorphogenesis) but could also harbor unique, uncharacterized roles.

**Figure 2:**
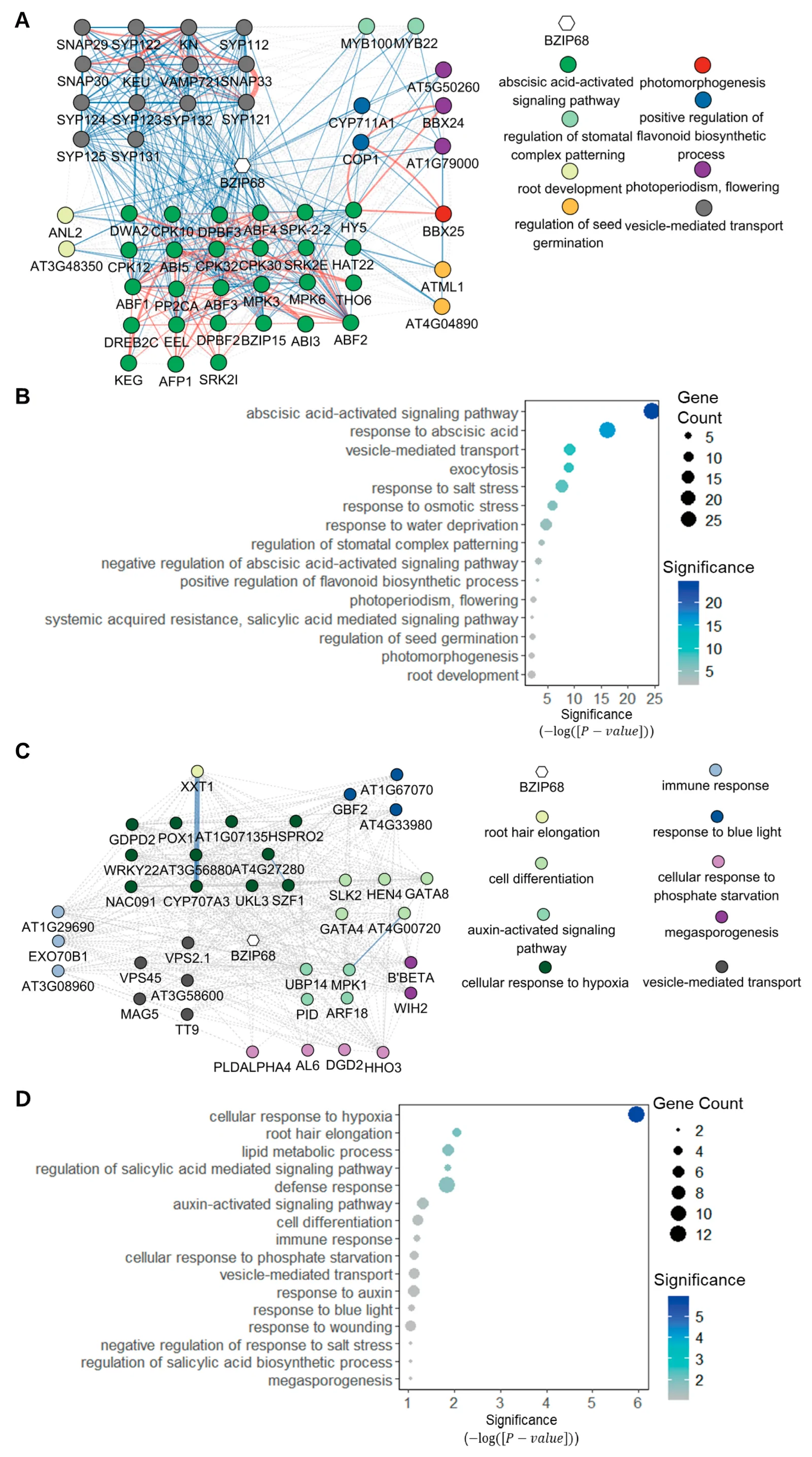
Functional annotation of bZIP68-interactome and bZIP68 co-expressed gene network. (**A**) bZIP68 interactome was represented. Each color filled in a circle indicates a representative functional annotation. Red line indicates physical interaction, blue line indicates predicted interaction, and gray dotted line indicates co-expressed relationship. (**B**) GO analysis on bZIP68 interactome. (**C**) bZIP68 co-expression network was represented. Each color filled in a circle indicates a representative functional annotation. Blue line indicates predicted interaction, and gray dotted line indicates co-expressed relationship. (**D**) GO analysis on bZIP68 co-expression network genes.

Then, we wanted to reveal the interactome and co-expressed gene network of bZIP68. The GeneMania-derived PPI interactome of bZIP68 contained 53 members, with eight functions identified through GO analysis (Figure 2A). Among them, 27 members of the interactome were associated with abscisic acid signaling pathways and 14 members were associated with vesicle-mediated transport (Figure 2A). Consistent with previous reports [14,60], photomorphogenesis-associated gene (BBX25) and flowering-associated genes were associated in the interactome as well (Figure 2A). Further GO analysis revealed that these members were enriched in ‘response to salt stress’, ‘response to osmotic stress’, and ‘root development’, which are in accordance with previous findings in *bzip68* mutants (Figure 2B) [12]. In addition, ‘regulation of seed germination’ was also enriched (Figure 2B). Co-expression network has 38 members with nine representative functions (Figure 2C). Eleven members were associated with genes involved in hypoxia and *XXT1*, a gene associated with root hair elongation, was strongly connected with these hypoxia-associated genes (Figure 2C). ‘Response to blue light’ and ‘megasporogenesis’ were also included in the network (Figure 2C). GO enrichment analysis of the 38 genes identified within the co-expression network revealed an overrepresentation of GO terms related to immune and wounding responses (Figure 2D). Also, auxin-activated signaling pathway-associated genes and cell differentiation-associated genes were enriched, explaining the growth finetuning effects under abiotic stress conditions described in previous reports (Figure 2D) [12]. These results indicate that our network analysis validates the previously identified roles of bZIP68 in photomorphogenesis, stress-mediated growth regulation, and flowering (Figure 2). Furthermore, these data implicate bZIP68 in modulating additional biological processes, including seed germination and defense responses (Figure 2).

### 3.2. bZIP68 Transcriptome and ChIP-Seq Analysis

**Figure 3:**
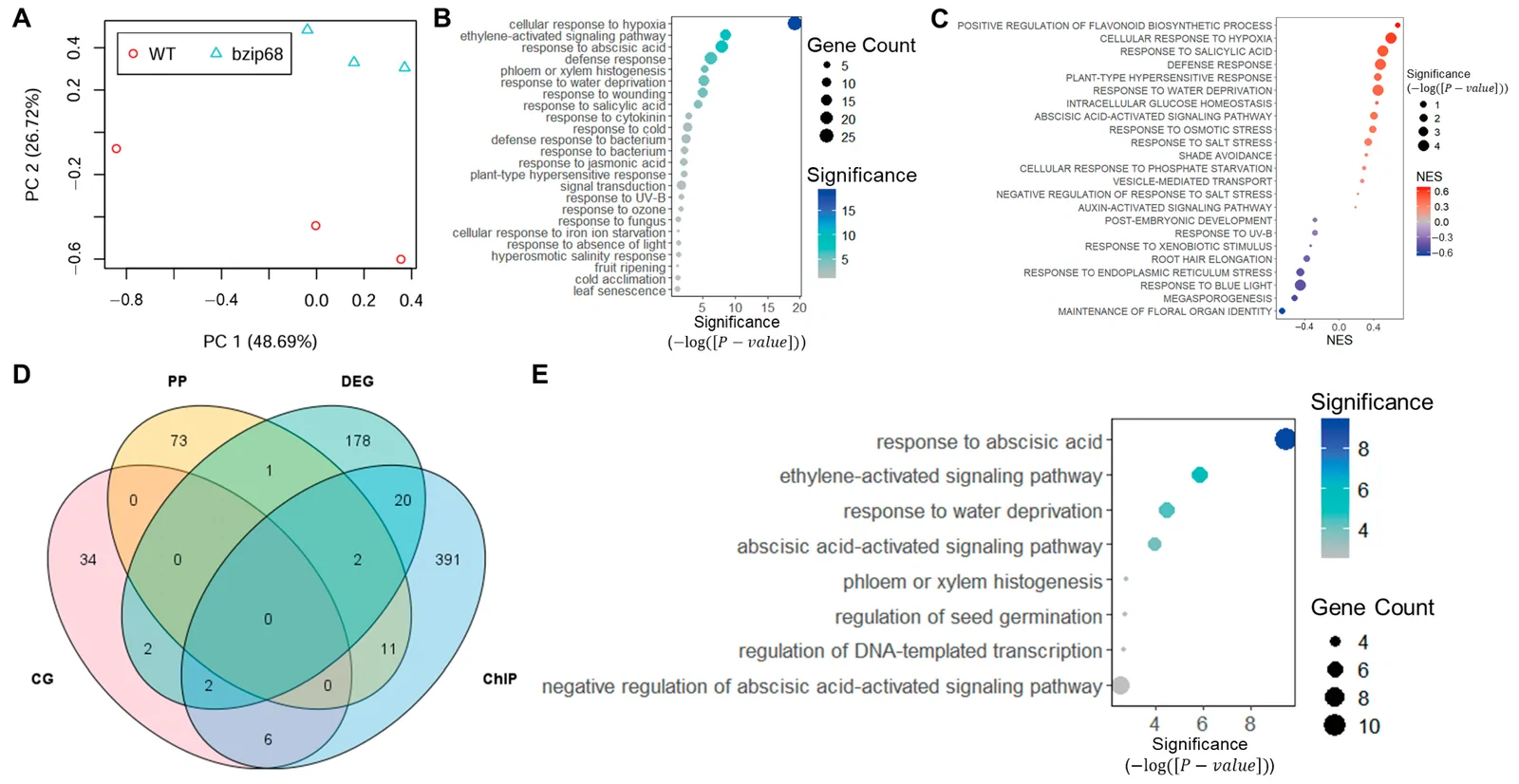
Analysis of the bZIP68-associated transcriptome and direct target genes. (**A**) PCA of the *bzip68* transcriptome. (**B**) GO analysis of DEGs derived from the *bzip68* transcriptome. (**C**) GSEA of the *bzip68* transcriptome. (**D**) Venn diagram representation of PPI network (PP), co-expressed gene network (CG), DEG, and ChIP-seq (ChIP) target genes of bZIP68. (**E**) GO analysis on bZIP68 direct target genes.

To analyze how bZIP68 modulates the transcriptome landscape, we re-analyzed previously published *bzip68* mutant transcriptomic datasets (Figure 3) [12]. Principal component analysis (PCA) revealed distinct transcriptomic profiles between wild-type and *bzip68* mutant (Figure 3A). We identified 205 DEGs and subsequently performed GO and GSEA analyses to annotate their biological functions (Figure 3B,C). Abiotic stress-related GO terms, such as ‘cellular response to hypoxia’ or ‘response to water deprivation’, were enriched in GO analysis (Figure 3B). In addition, light-correlated GO terms, such as ‘response to UV-B’ and ‘response to absence of light’, were identified (Figure 3B). Flower-associated genes, such as ‘fruit ripening’, were also enriched (Figure 3B). While defense-associated genes were enriched, terms associated with seed germination were not found (Figure 3B). From GO analysis, abiotic-stress-related terms (such as cellular response to hypoxia), light-related terms (such as response to blue light), flower-related terms (such as maintenance of floral organ identity), and defense-related terms (such as defense response) were enriched. In contrast, terms related to seed germination were not significantly enriched (Figure 3C). To further reveal the direct regulatory targets of bZIP68, we re-analyzed publicly available bZIP68 ChIP-seq data, identifying 24 high-confidence direct targets which indicates ChIP-seq enriched DEGs or network-included genes (Figure 3D). GO analysis of these targets revealed that genes involved in abiotic stress and flower development were highly enriched (Figure 3E).

### 3.3. Brief Functional Annotation of bZIP68

**Figure 4:**
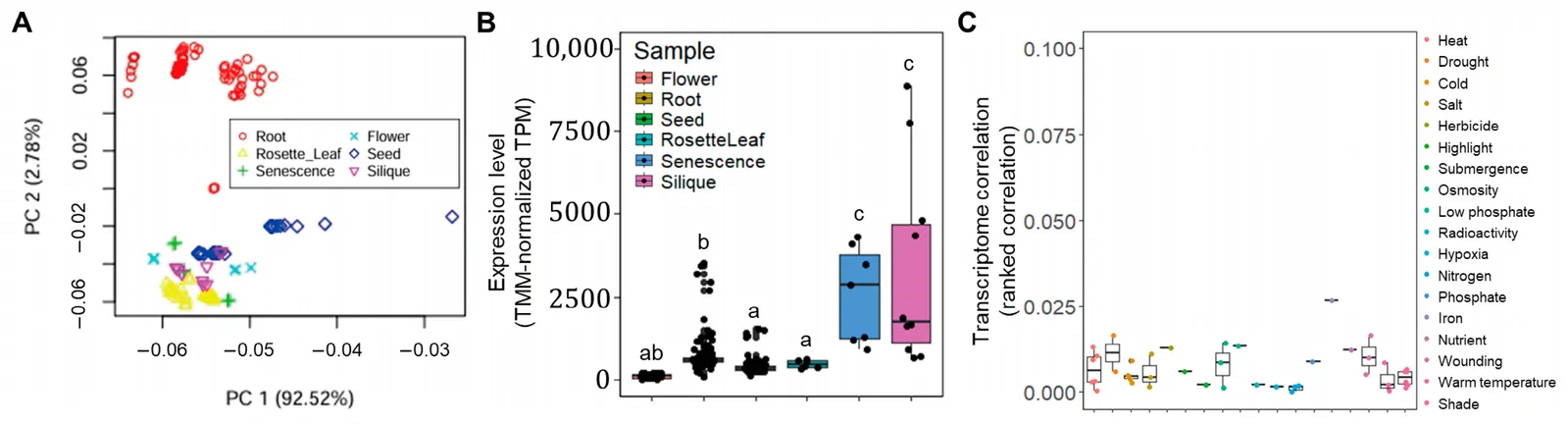
Re-analysis of the publicly available transcriptomic datasets to predict the physiological roles of bZIP68. (**A**) PCA of 385 transcriptomes consists of different tissues from wild-type plants. (**B**) Expression pattern of *bZIP68* from six tissues. Alphabets above boxes indicate statistical significance (*p* < 0.05). (**C**) Transcription correlation/association score of various conditions with *bzip68* mutant transcriptome. Association score was calculated as the absolute value and visualized.

Our comprehensive network and GO enrichment analyses implicate bZIP68 in diverse physiological processes, including abiotic stress regulation, flower-associated development, photomorphogenesis, defense responses, and seed germination (Figure 2 and Figure 3). Given the diversity of these predicted functions, we aimed to refine the putative function of bZIP68 by analyzing publicly available transcriptomes across various tissues and experimental conditions (Tables S1 and S2). To rule out developmental or tissue biases, we restricted our meta-analysis to transcriptomic datasets derived from 7- to 14-day-old seedlings and upper-ground tissues (Table S2). By analyzing 385 transcriptomes from six different tissues (Root, Rosette leaf, Senescent leaf, Flower, Seed, and Silique), we examined the spatial expression profile of *bZIP68* (Figure 4A,B). *bZIP68* was predominantly expressed in senescent leaves (this dataset includes abiotic-stress-accelerated senescent leaves) and siliques, with a relatively high expression observed in root tissue (Figure 4B). Furthermore, to predict the environmental conditions associated with bZIP68, we calculated transcriptome association scores comparing the *bzip68* mutant transcriptome to datasets spanning various environmental and stress conditions (Figure 4C; Table S2). However, none of the transcriptome datasets tested showed a high correlation with *bzip68* mutant transcriptomic landscape (Figure 4C). Taken together, these data suggest that bZIP68 function may be more pronounced during adult developmental stages rather than during seed germination.

### 3.4. Identification of bZIP68 Regulome

**Figure 5:**
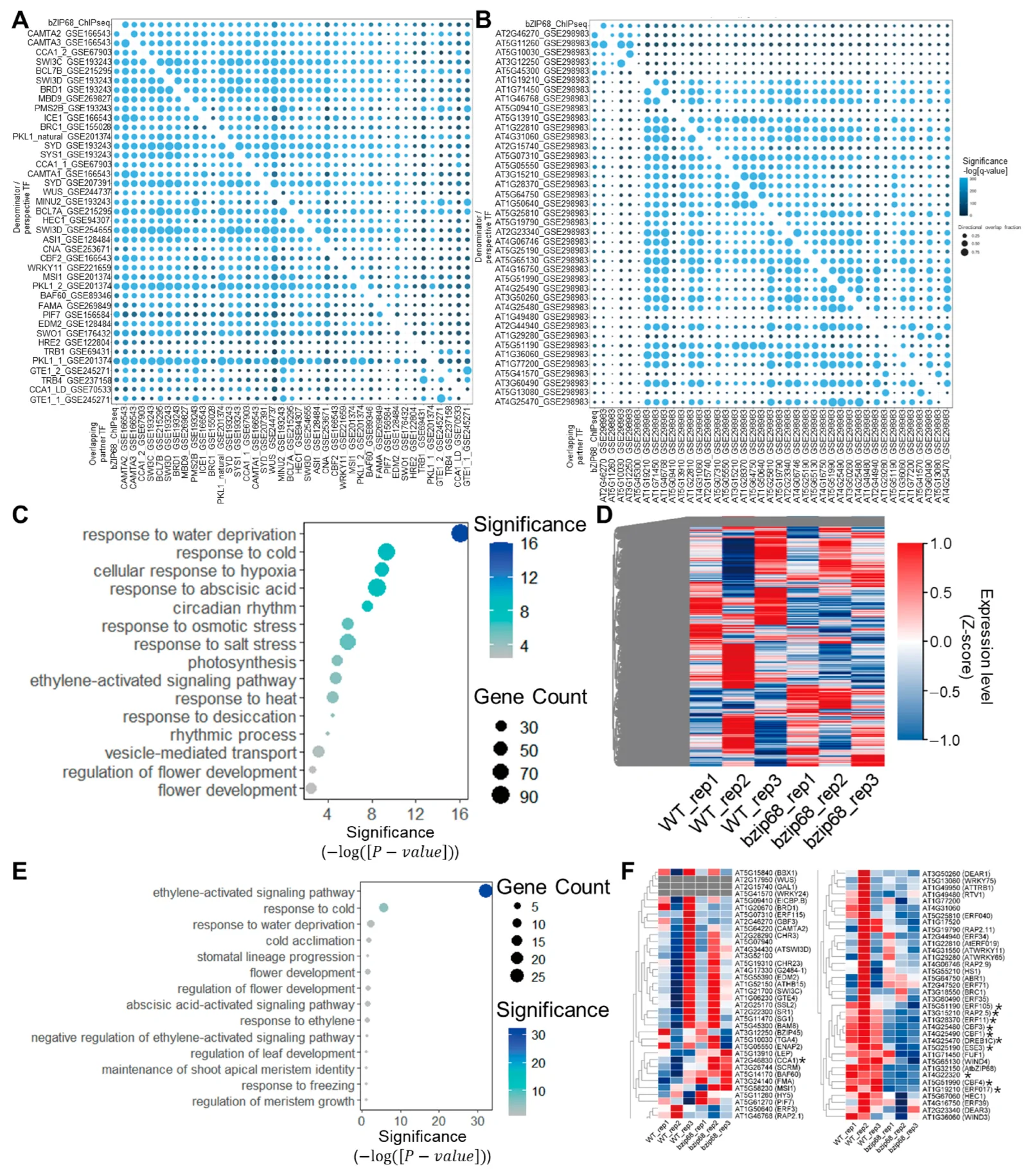
Identification and functional annotation of putative bZIP68 regulome. (**A**) Heatmap representation of comparative ChIP-seq analysis with bZIP68 ChIP-seq data. (**B**) Heatmap representation of comparative DAP-seq analysis with bZIP68 ChIP-seq data. (**A**,**B**) Only the top 40 samples are shown. Dot size indicates directional overlap fraction, while pale color indicates more significantly enriched. (**C**) GO analysis on target genes of the putative bZIP68 regulome. (**D**) Expression pattern of target genes of putative bZIP68 regulome in *bzip68* transcriptome. (**E**) GO analysis of putative bZIP68 regulome member genes. (**F**) Expression pattern of putative bZIP68 regulome member genes in *bzip68* transcriptome. Asterisk indicates the regulome genes showed differential expression by bzip68 mutation. (**D**,**F**) The color key for expression is shared. Expression level was represented with Z-score.

To reveal the regulome of bZIP68, we adapted all the available ChIP-seq and DAP-seq data from the public database (Table S3). A total of 1435 sequencing datasets were analyzed and compared with bZIP68 ChIP-seq data by concentrating on peak points and a 500 bp window (Figure 5). For ChIP-seq datasets, we considered biologically relevant experimental conditions, including the sampling stage (7–15 days) and whole-seedling samples, whenever possible. In contrast, DAP-seq datasets were analyzed without restricting the experimental conditions (Table S3). By analyzing ChIP-seq and DAP-seq datasets separately, we could identify 72 putative regulome members of bZIP68 (Figure 5A,B; Table 1). Among them, 57 members were transcription factors while 15 members were epigenetic factors (Table 1).

Next, we investigated the downstream targets of this bZIP68-mediated regulome. We defined a gene as regulome-target gene (RTG) if it contained a shared 300 bp window bound by at least 20 factors other than bZIP68. By setting 20 factors as a threshold, we could select RTGs with bZIP68 peak shared with at least 28.9% of putative regulome members. GO analysis of RTGs and profiling their expression pattern in *bzip68* transcriptome were conducted to reveal the direct regulatory contribution of bZIP68 on those regulome targets and functions (Figure 5C,D). RTGs were enriched with abiotic-stress-associated terms and flower-development-associated terms (Figure 5C). However, the expression profiles of these RTGs were not consistently affected in *bzip68* mutant (Figure 5D), suggesting that bZIP68 is associated with the regulation of gene expression through complex regulatory mechanisms. To reveal which physiological functions were associated with the putative regulome members, we performed GO analysis for these members (Figure 5E). Putative regulome members were enriched with abiotic-stress-associated terms and flower-associated terms (Figure 5E). Among them, the expression of 11 genes (*CCA1*, *ERF105*, *RAP2.5*, *ERF11*, *CBF3*, *CBF1*, *DREB1c*, *ESE3*, *AT4G23320*, *CBF4* and *ERF017*) was altered in *bzip68* mutant relative to the wild-type, suggesting that these genes are likely regulated directly or indirectly by bZIP68.

### 3.5. Identification of bZIP68 Core Regulome and Associated Gene Networks

**Figure 6:**
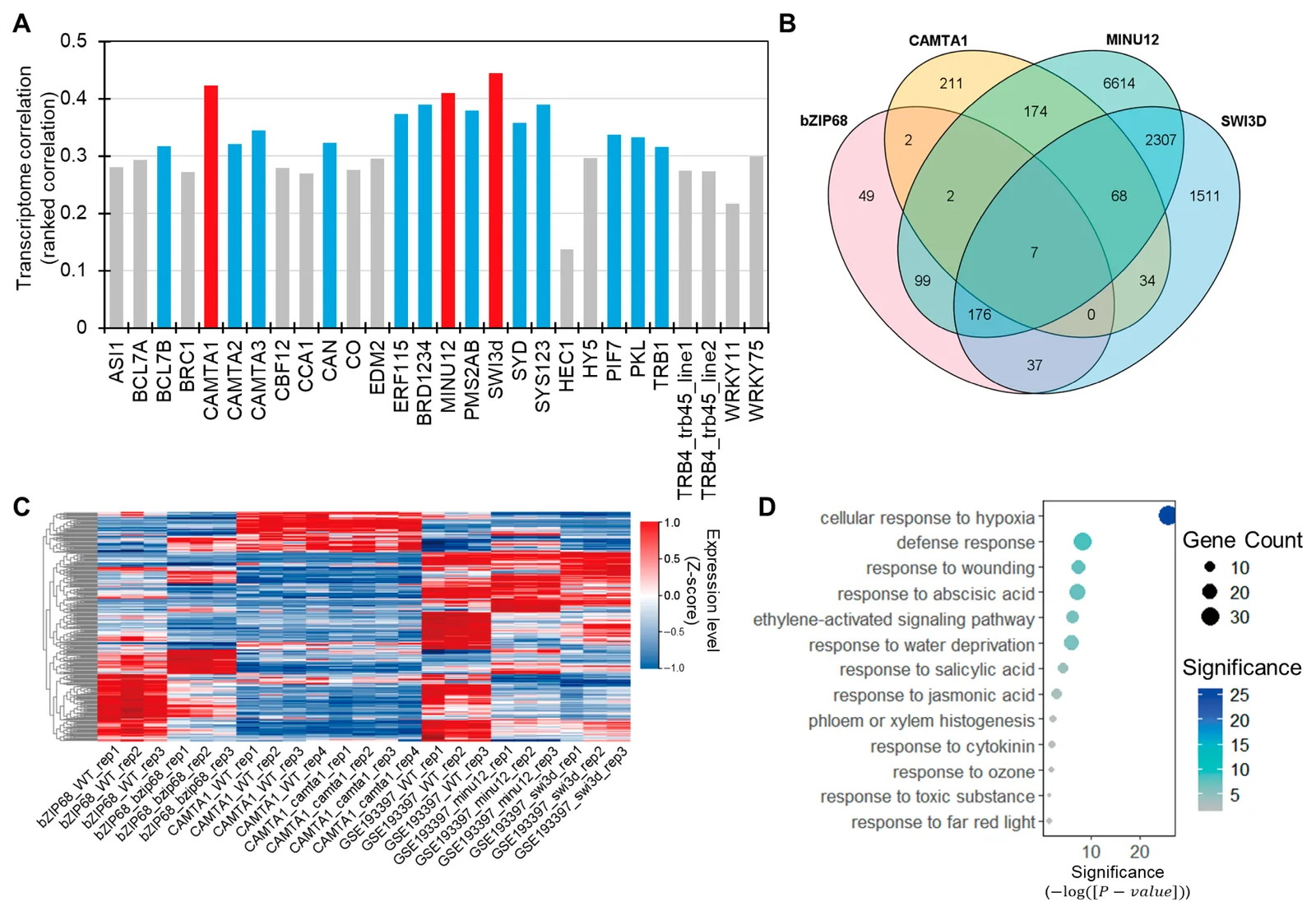
Identification of core regulome of bZIP68. (**A**) Association scores of putative regulome member mutant transcriptomes compared with *bzip68* mutant transcriptome. The absolute value of the ranked correlation was visualized. Association scores more than 0.3 were colored blue, while scores over 0.4 were colored red. CBF12 indicates *cbf1*; *cbf2*, BRD1234 indicates *brd1*; *brd2*; *brd3*; *brd4*, PMS2AB indicates *pms2a*; *pms2b*, SYS123 indicates *sys1*; *sys2*; *sys3*, and MINU12 indicates *minu1*; *minu2*. (**B**) Venn diagram of DEGs from core regulome transcriptome analysis. (**C**) Heatmap representation of 323 DEGs shared between *bzip68* and at least one core regulome member. Expression level was visualized in Z-score. (**D**) GO analysis of 323 DEGs was visualized.

To further define the regulatory mechanisms of bZIP68, we sought to identify a core regulome which exhibits transcriptional regulatory patterns consistent with bZIP68. We calculated transcriptome association scores by comparing *bzip68* mutant transcriptome profile against publicly available RNA-seq datasets for mutants of genes within the bZIP68-mediated regulome (Table 1 and Table S4). To minimize the effects of differences in experimental conditions, we focused on datasets with conditions comparable to those used for the *bzip68* mutant, including mock or mock-like treatments and shoot or whole-seedling samples collected from 6- to 14-day-old plants. The mutants of *BCL7B*, *CAMTA2*, *CAMTA3*, *CNA*, *ERF115*, *BRD1*, *PMS2B*, *SYD*, *SYS1*, *PIF7*, *PKL*, *TRB1*, *CAMTA1*, *MINU2*, and *SWI3D* displayed moderate association with the *bZIP68* mutant (Figure 6A). Among these, we defined CAMTA1, MINU2, and SWI3D as the core regulome of bZIP68 (bZIP68 core regulome), based on their transcriptome association scores exceeding 0.4. This threshold was selected as a data-driven operational cutoff corresponding to a natural separation within the upper tail of the observed score distribution. To identify the genes regulated by the bZIP68 core regulome, we performed a comparative transcriptomic analysis (Figure 6B). To capture a broader overview of the transcriptomic landscape, we applied a more permissive significance threshold (FDR < 0.05) to define DEGs. This approach identified 323 DEGs shared between *bzip68* and at least one core regulome member, thereby including genes showing partial associations with individual core regulome members (Figure 6B). The expression profiles of these DEGs in *bzip68* mutant were comparable to those observed in *minu1* and *swi3d* mutants, whereas *camta1* displayed subtle alterations in expression patterns (Figure 6C). Those DEGs were enriched with abiotic-stress-associated GO terms and phloem and xylem histogenesis, and defense response and light-associated GO terms (Figure 6D).

**Figure 7:**
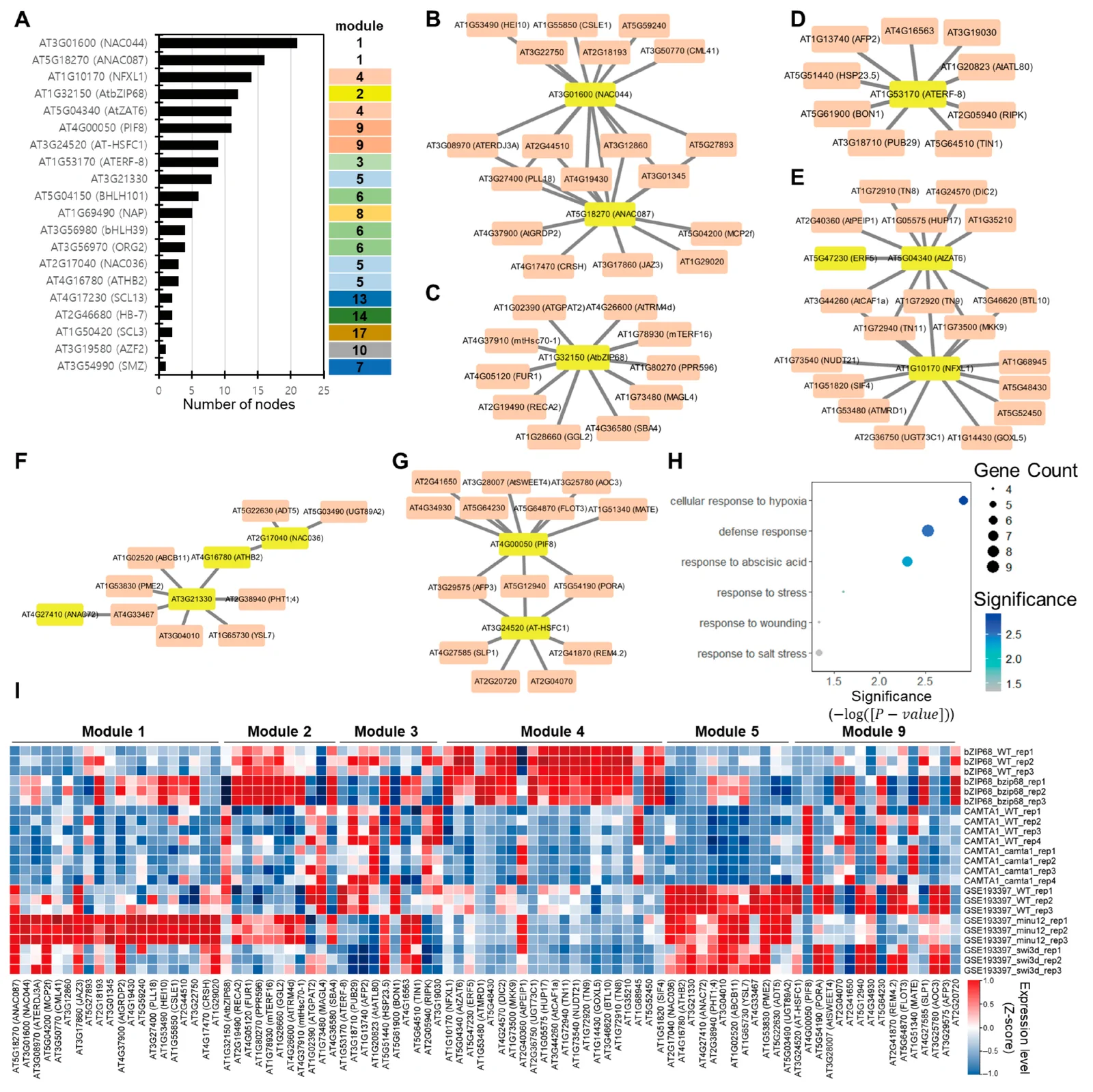
Potent regulatory network of bZIP68 core regulome. (**A**) GENIE3-identified modules with the number of nodes on the *x*-axis and regulator genes on the *y*-axis. (**B**) Representation of module 1. (**C**) Representation of module 2. (**D**) Representation of module 3. (**E**) Representation of module 4. (**F**) Representation of module 5. (**G**) Representation of module 9. (**B**–**G**) Yellow box indicates regulators while apricot box indicates target genes. (**H**) GO analysis on all the regulatory network genes. (**I**) Heatmap representation of expression patterns of the regulatory network genes.

To further reveal genes involved in the regulatory cascade of the core regulome, we performed GENIE3-based transcriptome module analysis using the 323 DEGs identified above (Figure 6 and Figure 7). In total, 13 modules were identified to be enriched with potent regulatory nodes. Among those modules, six of them included more than seven potent regulatory nodes (Figure 7A). Module 1 contained two regulators (NAC044, ANAC087), and 18 target genes (Figure 7B). Module 2 represented a bZIP68 regulatory module, suggesting an auto-regulatory circuit involving bZIP68 (Figure 7C). Module 3 was centered on ERF-8 and comprised nine target genes (Figure 7D). Module 4 contained two main regulators (ZAT6 and NFXL1) and one less-connected regulator (ERF6) (Figure 7E). Module 5 included four regulators, one of which was AtHB2, and its target genes were associated with flavonoid biosynthesis (Figure 7F). Module 9 included PIF8 and HSFC1 as regulators, which had 14 target genes (Figure 7G). GO analysis showed that genes in these modules were enriched in terms associated with abiotic responses and defense responses (Figure 7H). The expression profiles of the module-associated genes showed that modules 1, 2, 3, and 9 exhibited greater changes in expression than the other modules (Figure 7I). These module analyses provide further support for the involvement of bZIP68 in abiotic stress responses and photomorphogenesis, but they did not provide clear evidence for its role in flowering regulation.

### 3.6. Flower-Development-Associated Phenotype of bzip68 Mutants

**Figure 8:**
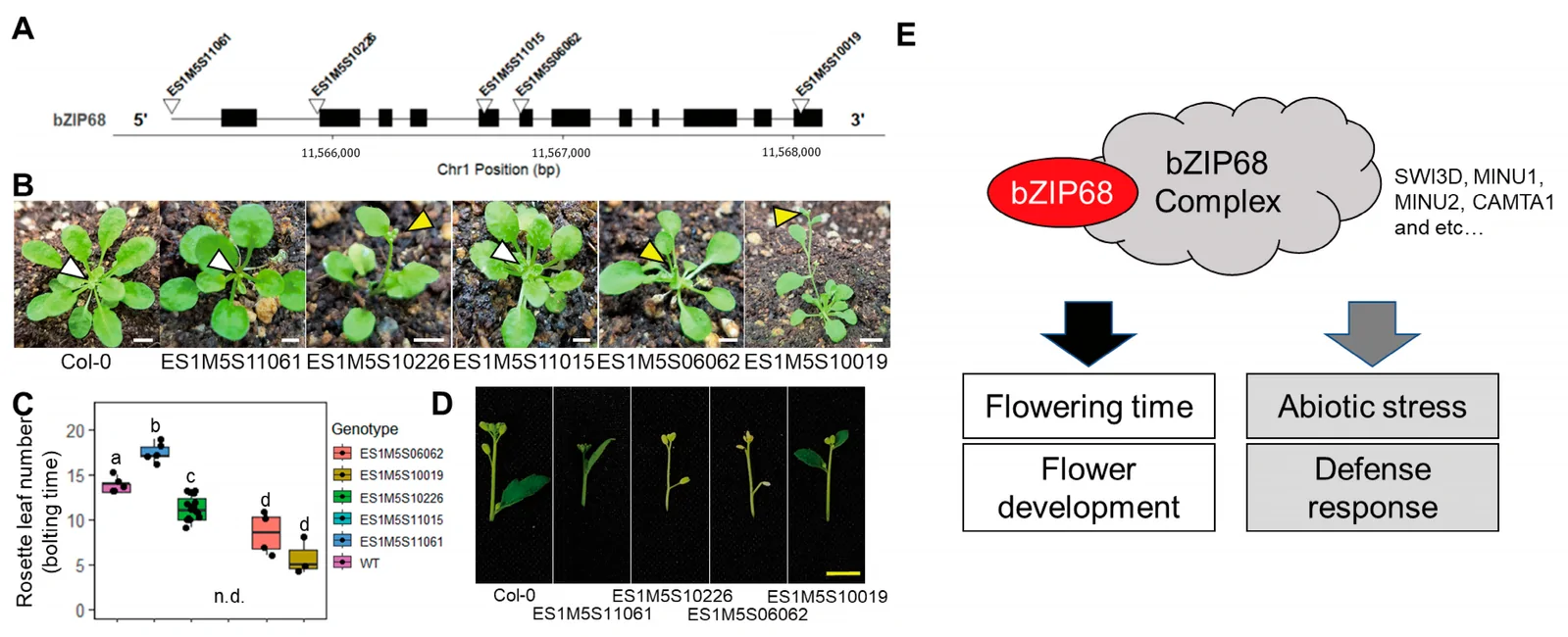
Summary of *bzip68* mutant analysis. (**A**) The mutation locations of the five HEM lines we used are visualized. (**B**) Flowering time phenotype of five HEM lines for bZIP68 was visualized. The picture was taken at 63 days after germination (DAG). White arrowhead indicates vegetative leaves while yellow arrowhead indicates floral buds. Scale bar = 1 cm. (**C**) Bolting time was visualized with boxplot with dots. Bolting time was measured by rosette leaf number of the bolting stage and dots indicate rosette leaf number of each sample. Characters above the boxes indicate statistical significance by Tukey’s test, *p* < 0.05. (**D**) Flower morphology was visualized by representative picture. Scale bar = 5 mm. (**E**) Suggested model through this study. Black arrow indicates physiological function with phenotype evidence from this study, while gray arrow indicates suggested function from bioinformatic analysis.

Our comprehensive bioinformatic analyses indicated that bZIP68 functions in abiotic stress regulation, flower developmental processes, and defense responses (Figure 2, Figure 3, Figure 4, Figure 5, Figure 6 and Figure 7). Given that the hint for bZIP68 modulating flowering time has been previously reported [12], we sought to further validate this phenotype. We fetched five HEM lines that were already analyzed with full SNP data [61]. To avoid the potential second site effects, we selected five independent HEM lines which holds homozygous mutation in *bZIP68* gene region with the following criteria (Table 2; Figure 8A): (1) the presence of a mutation within the bZIP68 gene region with at least a moderate predicted impact; (2) the presence of an EMS-induced mutation specifically within *bZIP68*; and (3) no other shared mutations across the five lines outside of the *bZIP68* locus. When we grew five lines with Col-0 in long-day conditions, we observed that all of them had altered flowering times accompanied by abnormal flower morphology compared to Col-0 (Figure 8B–D and Figure S1; Table 3). In summary, our integrative bioinformatic approach consistently predicted the role of bZIP68 in flowering phenotype, which was subsequently confirmed through the physiological characterization of these independent mutant lines (Figure 8B,C).

## 4. Discussion

Our study focused on the *A. thaliana* transcription factor bZIP68, for which previous reports have suggested limited physiological roles [12,14]. In particular, the available evidence for its involvement in flowering regulation has been relatively subtle [12]. To further characterize the potential functions of bZIP68, we performed a phylogenetic analysis of bZIP68 and its homologs. This analysis revealed that bZIP68 and its closest homologs share conserved functions while also exhibiting distinct functional characteristics, suggesting that bZIP68 has evolved specialized roles in specific physiological processes (Figure 1) [14]. PPI network and co-expression network analyses suggested that bZIP68 may contribute to multiple physiological processes, including seed germination, flowering, abiotic stress responses, photomorphogenesis, and defense responses (Figure 2). Similar functional associations were also identified among genes potentially targeted by bZIP68 (Figure 3). However, the relatively high expression of *bZIP68* in roots, senescing leaves, and siliques suggests that seed germination may be a less likely primary target of bZIP68-mediated physiological regulation (Figure 4B). Furthermore, none of the environmental or stress-treatment transcriptomic datasets showed a strong association with *bzip68* mutant transcriptome, making it difficult to establish a specific role for bZIP68 in responses to particular environmental stresses (Figure 4C). 

Among the putative regulome members, several core regulome members showed relatively strong associations with *bzip68* mutant transcriptome (Figure 5 and Figure 6; Table 1). Although these core regulome members were highly associated with bZIP68 at both ChIP-seq and transcriptomic levels, these associations does not necessarily imply direct regulatory interactions. Instead, the observed transcriptomic similarities may be mediated indirectly through secondary or tertiary regulatory networks. This possibility is supported by the limited overlap in DEGs between *bzip68* and *camta1* mutants. The core regulome members were selected based on their correlation scores, which captures genome-wide directional trends in log_2_FC values across genes and do not require a large number of statistically significant shared DEGs (Figure 6B,C). Therefore, the identified core regulome should be considered a putative regulatory network rather than a set of confirmed direct regulators of bZIP68. GO analysis of the putative downstream components of the core regulome did not provide a clear mechanistic explanation for the flowering-time and developmental regulations. However, the enrichment of GO terms related to vascular development suggests a potential connection to flowering regulation, since these processes have been associated with developmental phase transitions. 

Our comprehensive bioinformatic analyses revealed that genes associated with abiotic stress responses, flower development, and defense responses were consequently enriched as a potential role of the bZIP68 (Figure 8E). Consistent with these predictions, flowering time and developmental phenotypes were altered in five independent *bzip68* mutant lines (Figure 8B and Figure S1). These independent mutant phenotypes provide experimental support for the role of bZIP68 in flowering-time and developmental regulation. 

The direct interactome analysis indicated that HY5 directly interacts with bZIP68, whereas COP1 was indirectly associated with the bZIP68 interactome (Figure 2A). Both proteins are highly pleiotropic regulators whose functions extend beyond photomorphogenesis to include seed germination, flowering-time regulation, abiotic stress responses, and plant immunity [62,63,64,65,66,67]. HY5 was identified as a putative bZIP68 regulome member, and its mutant transcriptome showed a relatively weak association with *bzip68* mutant transcriptome (0.297 in association score; Table 1; Figure 6A). Thus, HY5 may be part of the bZIP68 regulome but may play a relatively minor role, potentially contributing photomorphogenic functions or fine-tuning broader bZIP68-mediated processes. In the case of COP1, a direct interaction between COP1 and bZIP68 was not detected in the PPI interactome (Figure 2A). However, the interaction between bZIP68 and HY5, together with the interactions of HY5 with BBX24 and BBX25, suggests that COP1 may be connected to the bZIP68 complex (Figure 2A) [60,68]. COP1-interacting factors, including BBX24, BBX25, and HY5, may contribute to the representation of COP1-associated functions within the bZIP68 complex (Figure 2A). HY5 positively regulates photomorphogenesis, whereas BBX24 and BBX25 generally attenuate HY5-dependent photomorphogenic responses. These relationships are consistent with the possibility that HY5 and its associated factors contribute to the bZIP68 regulatory network associated with photomorphogenesis (Figure 2A and Figure 6A) [60,69].

Five independent *bzip68* mutant lines showed altered flowering time phenotypes, although the putative downstream pathways did not include genes specifically associated with flower development (Figure 6D). Although the five independent HEM lines carried distinct mutations in *bZIP68* genomic region without shared mutation (Figure 8A), we cannot completely exclude the possibility that background mutations contributed to the observed flowering phenotypes. Nevertheless, a previous study also reported that *bzip68* mutations can induce an early-flowering phenotype in an allele-dependent manner [12]. Therefore, the possibility that bZIP68 contributes to flowering regulation appears more plausible than an explanation based on background mutations in HEM lines. In this context, bZIP68 appears to share regulatory target space with CAMTA1 and with distinct SWI/SNF chromatin-remodeling modules represented by MINU2 and SWI3D (Figure 7 and Figure 8C). Recently, the SYD-associated SWI/SNF (SAS) complex containing SWI3D and the MINU1/2-associated SWI/SNF (MAS) complex containing MINU1/2 were shown to be negatively associated with chromatin accessibility regulation [70]. CAMTA1 is also known to regulate cold-/drought-stress responses and calcium-responsive signaling [71]. These findings suggest the possibility that flowering time may be fine-tuned through interaction between bZIP68 and SWI3D- or MINU1/2-associated regulatory networks. Overall, our results provide substantial evidence supporting a contribution of bZIP68 to flowering-time regulation, although further studies using complementation lines and targeted bZIP68 mutants will be necessary to establish this role and clarify the underlying molecular mechanisms.

Our result also suggested that bZIP68 may play a physiological role in defense responses (Figure 8C). We found that *wrky75* mutant transcriptome showed a relatively high association score (0.300) with *bzip68* mutant transcriptome (Figure 6A). In *A. thaliana*, WRKY75 has been implicated in defense against necrotrophic fungi through JA-ET dependent manner [72]. Thus, *bzip68* mutation may alter a transcriptional state that partially overlaps with the WRKY75-dependent defense program (Figure 6A). The transcriptomic datasets of CAMTA family transcription factors mutants (*camta1*, *camta2*, and *camta3*) also showed relatively high association scores with *bzip68* transcriptome (Figure 6A). CAMTA proteins are known to regulate pipecolic acid biosynthesis and the SA-related defense programs [73,74]. In addition, EDM2 (0.295) and ASI1/IBM2 (0.280) showed relatively high association scores. These factors regulate the functionality of *RPP7* transcripts and contribute to resistance against *Hyaloperonospora arabidopsidis* [75,76]. Although EDM2 and ASI1 enhance NLR-mediated specific immunity, they can also negatively regulate basal SA-dependent immunity [77]. ERF115 showed an association score of 0.3731 and is known to function in wound-induced regeneration in response to nematode infection [78,79]. HY5 also showed an association score of 0.297 and is known to regulate light-responsive defense responses [67]. Taken together, these associations suggest that the bZIP68-associated regulatory network may contribute to defense responses through complex regulatory pathways involving CAMTAs, EDM2-ASI1, WRKY75, ERF115, and HY5. In this study, we elucidated the physiological functions of bZIP68 through a comprehensive integrative bioinformatic approach, which was further validated by the phenotypic characterization of five independent HEM lines demonstrating altered flowering time (Figure 8). The additional roles of bZIP68 suggested by our bioinformatic predictions remain to be explored.

## 5. Conclusions

In this study, we established an integrative bioinformatic pipeline to predict the potential physiological functions and regulatory networks of genes. Using this approach, we predicted that bZIP68 is involved in flowering, abiotic stress responses, and defense-related responses, together with its putative regulome members. Although the involvement of bZIP68 in abiotic stress responses and photomorphogenesis has been previously shown, its role in flowering modulation has been unclear. Here, we provide experimental evidence supporting a role for bZIP68 in flowering regulation based on the analysis of five independent HEM lines. Taken together, our comprehensive bioinformatic analyses and phenotypic evaluation provide new insights into the physiological functions of bZIP68 and identify the potential roles of its regulatory partners, providing a foundation for future investigation. 

## Figures and Tables

**Table 1:** List of putative bZIP68 regulome members. TF = Transcription factor, EF = Epigenetic factor, NC = Nuclease, TBF = Telomere binding factor.

Factor Name	AGI	Origin Data	Molecular Type	Experiment
bZIP68	AT1G32150	GSE133900	TF	ChIP-seq
CAMTA1	AT5G09410	GSE166543	TF	ChIP-seq
CAMTA2	AT5G64220	GSE166543	TF	ChIP-seq
CAMTA3	AT2G22300	GSE166543	TF	ChIP-seq
CBF2	AT4G25470	GSE166543	TF	ChIP-seq
HRE2	AT2G47520	GSE122804	TF	ChIP-seq
ASI1	AT5G11470	GSE122804	EF	ChIP-seq
EDM2	AT5G55390	GSE122804	TF; EF	ChIP-seq
BRC1	AT3G18550	GSE155028	TF	ChIP-seq
PIF7	AT5G61270	GSE156584	TF	ChIP-seq
SWO1	AT4G17330	GSE176432	EF	ChIP-seq
BRD1	AT1G20670	GSE193243	EF	ChIP-seq
MINU2	AT5G19310	GSE193243	EF	ChIP-seq
PMS2B	AT3G52100	GSE193243	NC	ChIP-seq
SWI3C	AT1G21700	GSE193243	EF	ChIP-seq
SWI3D	AT4G34430	GSE193243; GSE254655	EF	ChIP-seq
SYD	AT2G28290	GSE193243, GSE207391	EF	ChIP-seq
SYS1	AT5G07940	GSE193243	EF	ChIP-seq
MSI1	AT5G58230	GSE201374	WD40	ChIP-seq
PKL	AT2G25170	GSE201374	EF	ChIP-seq
BCL7A	AT4G22320	GSE215295	EF	ChIP-seq
BCL7B	AT5G55210	GSE215295	EF	ChIP-seq
WRKY11	AT4G31550	GSE221659	TF	ChIP-seq
CO	AT5G15840	GSE222657	TF	ChIP-seq
TRB4	AT1G17520	GSE237158	TBF	ChIP-seq
WUS	AT2G17950	GSE244737	TF	ChIP-seq
GTE4	AT1G06230	GSE245271	EF	ChIP-seq
CNA	AT1G52150	GSE253671	TF	ChIP-seq
FAMA	AT3G24140	GSE269849	TF	ChIP-seq
CCA1	AT2G46830	GSE67903, GSE70533	TF	ChIP-seq
TRB1	AT1G49950	GSE69431	TBF	ChIP-seq
BAF60	AT5G14170	GSE89346	EF	ChIP-seq
HEC1	AT5G67060	GSE94307	TF	ChIP-seq
ICE1	AT3G26744	GSE166543	TF	ChIP-seq
GBF3	AT2G46270	GSE298983	TF	DAP-seq
HY5	AT5G11260	GSE298983	TF	DAP-seq
TGA4	AT5G10030	GSE298983	TF	DAP-seq
TGA6	AT3G12250	GSE298983	TF	DAP-seq
BAM8	AT5G45300	GSE298983	TF	DAP-seq
ACRE2	AT1G19210	GSE298983	TF	DAP-seq
FUF1	AT1G71450	GSE298983	TF	DAP-seq
RAP2.1	AT1G46768	GSE298983	TF	DAP-seq
CAMTA1	AT5G09410	GSE298983	TF	DAP-seq
LEP	AT5G13910	GSE298983	TF	DAP-seq
AtERF019	AT1G22810	GSE298983	TF	DAP-seq
ERF015	AT4G31060	GSE298983	TF	DAP-seq
GAL1	AT2G15740	GSE298983	TF	DAP-seq
ERF115	AT5G07310	GSE298983	TF	DAP-seq
VFP5	AT5G05550	GSE298983	TF	DAP-seq
ERF4	AT3G15210	GSE298983	TF	DAP-seq
ERF11	AT1G28370	GSE298983	TF	DAP-seq
ABR1	AT5G64750	GSE298983	TF	DAP-seq
ERF3	AT1G50640	GSE298983	TF	DAP-seq
TINY	AT5G25810	GSE298983	TF	DAP-seq
RAP2.11	AT5G19790	GSE298983	TF	DAP-seq
DEAR3	AT2G23340	GSE298983	TF	DAP-seq
RAP2.9	AT4G06746	GSE298983	TF	DAP-seq
ESE3	AT5G25190	GSE298983	TF	DAP-seq
WIND4	AT5G65130	GSE298983	TF	DAP-seq
ERF39	AT4G16750	GSE298983	TF	DAP-seq
CBF4	AT5G51990	GSE298983	TF	DAP-seq
CBF1	AT4G25490	GSE298983	TF	DAP-seq
DEAR1	AT3G50260	GSE298983	TF	DAP-seq
DREB1A	AT4G25480	GSE298983	TF	DAP-seq
RTV1	AT1G49480	GSE298983	TF	DAP-seq
ERF34	AT2G44940	GSE298983	TF	DAP-seq
WRKY65	AT1G29280	GSE298983	TF	DAP-seq
ERF105	AT5G51190	GSE298983	TF	DAP-seq
TG	AT1G36060	GSE298983	TF	DAP-seq
AT1G77200	AT1G77200	GSE298983	TF	DAP-seq
WRKY24	AT5G41570	GSE298983	TF	DAP-seq
ERF035	AT3G60490	GSE298983	TF	DAP-seq
WRKY75	AT5G13080	GSE298983	TF	DAP-seq
CBF2	AT4G25470	GSE298983	TF	DAP-seq

**Table 2:** List of putative bZIP68 mutants.

Line Number	POS	ORIG	ALT	Location	Direct Effect	Potent Functional Effect
ES1M5S11061	11,565,302	C	T	Promoter	CACGTC into TACGTC	Lower transcript level of *BZIP68*
ES1M5S10226	11,565,933	G	A	1st Intron	possible alteration of 3′ splice-site recognition or splicing efficiency	Lower transcript level or altered form of 5’UTR of *BZIP68*
ES1M5S11015	11,566,660	C	T	5th Exon	Pro87Ser	Potential PTM or PPI interference
ES1M5S06062	11,566,818	C	T	6th Exon	Ser110Phe	Potential PTM or PPI interference
ES1M5S10019	11,568,034	G	A	12th Exon	Glu328Lys	bZIP helix/dimer properties or altered redox-regulation

**Table 3:** Phenotype observation of putative bZIP68 mutants.

Genotype	Flowering Time	INFLORESCENCE SIZE	Flower Number Per Inflorescence	Apical Dominance	Plant Size
ES1M5S11061	Late flowering	Small	Fewer	Normal	WT level
ES1M5S10226	Early flowering	Small	Fewer	Normal	Slight dwarfism
ES1M5S11015	Late flowering (not observed)	n.d.	n.d.	n.d.	WT level
ES1M5S06062	Early flowering	Small	Fewer	Normal	WT level
ES1M5S10019	Early flowering	Small	Fewer	Lost	Dwarfism

## Data Availability

All the data analyzed is presented in Supplementary Tables (Tables S1–S5).

## Supplementary Materials

The following supporting information can be downloaded at https://www.mdpi.com/article/10.3390/biology15171525/s1, Figure S1: Flowering time phenotype of five HEM lines for bZIP68. Picture was taken in 83 DAG; Table S1: List of transcriptomes to analyze tissue-specific expression of bZIP68; Table S2: List of transcriptomes to analyze environmental stimulus or stress association with *bzip68* mutant transcriptome; Table S3: List of ChIP-seq and DAP-seq data analyzed in this study; Table S4: List of transcriptomes to analyze regulome association with *bzip68* mutant transcriptome.; Table S5: Expression pattern of the RTGs in bzip68 mutant transcriptome.

## Abbreviations

The following abbreviations are used in this manuscript:

TF	Transcription Factors
EF	Epigenomic Factors
NC	Nuclease
TBF	Telomere Binding Factor
PPI	Protein Protein Interaction
HEM	Homozygous EMS Mutant
DEG	Differentially Expressed Genes
FDR	False Discovery Rate
EASE	Expression Analysis Systematic Explorer
GSEA	Gene Set Enrichment Analysis
TPM	Transcript Per Million
