# Supplementary material for: Arabidopsis bZIP68 Modulates Flowering Time Potentially with Chromatin and Stress-Responsive Regulators
Source: Biology (Basel). 2026 Sep 3;15(17):1525. doi: 10.3390/biology15171525 (PMC13564689; doi:10.3390/biology15171525)
Supplement: Supplementary file 1 [file biology-15-01525-s001.zip › Supplementary Figure.pdf]

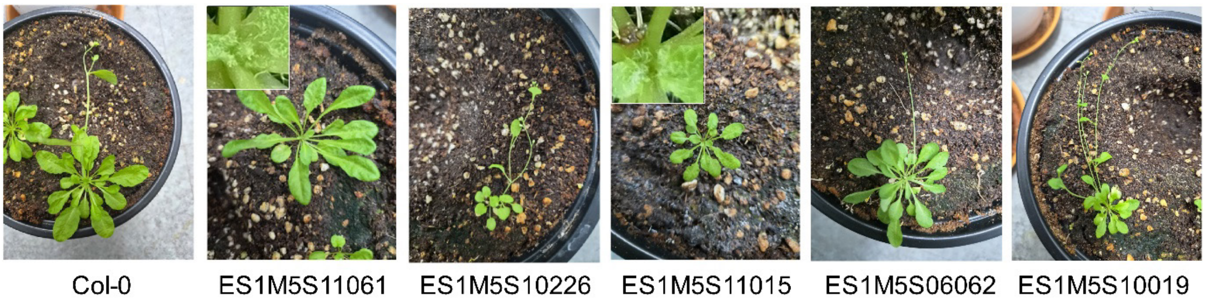

Figure S1. Flowering time phenotype of five HEM lines for bZIP68. Picture was taken in 83 DAG.
